# Profile of sexuality and symptoms of lower urinary tract in non-institutionalized elderly

**DOI:** 10.1590/S1677-5538.IBJU.2019.0162

**Published:** 2020-02-20

**Authors:** Khaled Ahmed Taha, Flavio Trigo Rocha, Lisias Castilho

**Affiliations:** 1 Hospital Augusto de Oliveira Camargo IdaiatubaSP Brasil Hospital Augusto de Oliveira Camargo, Idaiatuba, SP, Brasil; 2 Hospital Sirio Libanes Centro de Incontinência Urinária São PauloSP Brasil Centro de Incontinência Urinária, Hospital Sirio Libanes, São Paulo, SP, Brasil; 3 Universidade de São Paulo Faculdade de Medicina São PauloSP Brasil Faculdade de Medicina, Universidade de São Paulo, Urologia, São Paulo, SP, Brasil; 4 Faculdade de Medicina de Jundiai JundiaíSP Brasil Faculdade de Medicina de Jundiai, Jundiaí, SP, Brasil

**Keywords:** Women, Lower Urinary Tract Symptoms, Erectile Dysfunction

## Abstract

**Introduction:**

Urinary or sexual dysfunction in the elderly are underreported. However, they are highly prevalent. This study aims to identify the prevalence of these conditions.

**Objective:**

The aim is to carry out an investigation in non-institutionalized individuals over 60 years of age, to obtain data on its sexual and urinary health in São Paulo, Campinas, Santo André and Londrina.

**Results:**

6.000 questionnaires were distributed, and 3425 were included in the study, for the analysis of the questionnaires separately. In relation to ADAM, 92% of the 1385 evaluated were suspicious of androgen deficiency (ADAM). As for the male sexual function, it was observed 37% of premature ejaculation. As for the female sexual function, 1300 (74%) did not practice sexual intercourse and the main reasons were: lack of partner and lack of sexual desire. In addition, 988 (78%) of women who had no sexual intercourse responded that they didn't want sex and, more importantly, about 22% of them would like to have sexual intercourse. International prostate symptom score (IPSS) showed gradual worsening of urinary symptoms with increasing age, being the most prevalent: nocturia and urinary urgency. As for the female IPSS, we noted that even after 80 years, the majority have mild symptoms related to voiding dysfunction; with increasing age there is a gradual increase in the result of the IPSS.

**Conclusion:**

Due to the large number of sexual and urinary disorders found, we recommend the improvement in health conditions, promoting a better quality of life in the elderly.

## INTRODUCTION

### Sexual Function

The definition of erectile dysfunction (ED) was proposed in 1992, during a conference on impotence, being that the inability of achieving or maintaining an erection for a satisfying sexual relation.

Men with hypogonadism show few symptoms, and are frequently not diagnosed, denied by the patient and not evidenced by the doctor, affecting more than 10% of population (1). In a survey by Hospital das Clinicas in São Paulo, individuals submitted to prostate cancer screening showed a global prevalence of ED of 66% (2).

Sexual Disfunction affects around 152 million of men worldwide. In 2025, around 322 million of men are estimated to present ED. The direct costs with treatment in the United States (US) are estimated in 400 million dollars yearly (3).

Premature ejaculation (PE) is the second most prevalent disorder in men, with indicators ranging between 26 and 31%; little variation is found among different age groups, despite being more frequent in young men (4).

### Urinary Function

Data from the National and Nutrition Examination suggest that low urinary tract symptoms (LUTS) and benign prostatic hyperplasia (BPH) are common in men over 30, increasing with age (5). Over a follow-up period of 42 months it was observed an increase of the international prostate symptom score (IPSS) values in relation to moderate and major symptoms from 33% to 49% (6). Urinary retention is the final stage of BPH, occurring in 6.8 cases per 1000 individuals, increasing according to age, prostatic size and severity of the symptoms (7).

A method of great value in evaluating micturition dysfunction is the application of the American Urological Association-7 (AUA-7) questionnaire, initially used to assess emptying symptoms and later renamed IPSS. After that, it was considered the score of the life quality of the specific disease. It was also validated to the Portuguese language (8).

The IPSS was initially used to assess urinary symptoms in men with BPH. However, it became evident that IPSS was not specific to disease or gender. Therefore, the validation and use of the IPSS in women are as good as they are in men (9).

According to the National Kidney and Urologic Disease Advisory Board, urinary incontinence (UI) affects 13 million Americans, having an elevated frequency among elderly people (1035%), representing US$ 11 billion from the government expenses (10).

It is estimated that 50 million people worldwide suffer from UI, being that more common in women, affecting up to 50% of them at some point in life (11). In Brazil, it is estimated that up to 23% of the female population is incontinent, and, in elderly women, this prevalence can vary between 8 to 35% (12).

In 2002, the International Society of Continence established overactive bladder (OB) as a syndrome characterized by symptoms of urgent urination, with or without incontinence, usually with nocturia and increase of urinary frequency, with a prevalence of 34 million of individuals, most of them elderly people, with costs of US$ 12 billion per year.

## OBJECTIVE

To conduct an investigation of a non-institutionalized elderly population, of both sexes, in order to obtain data related to the prevalence of sexual and urinary dysfunction.

## MATERIALS AND METHODS

We assessed the prevalence of sexual and urinary dysfunction of these individuals by means of a validated questionnaire or in validation stage (e.g ADAM questionnaire).

It was performed an active search of respondents through phone books of the cities, to ensure that these elderly individuals were a reliable sample of the Brazilian population not linked to any medical service. There was home delivery of these questionnaires, with explanation and signature of the consent form. After seven days, the researches collected these documents.

Inclusion criteria: male or female, with age equal to or above sixty, non-institutionalized, capable of comprehending the study

Exclusion criteria: the ones who had difficulties in understanding the goals, illiterate people or unable to answer without help and questionnaires answered incorrectly.

Applied questionnaires: income (income assessment according to the number of monthly minimum wage received), androgen deficiency in the aging male (ADAM) questionnaire (elderly screening questionnaire for evaluation of androgenic disorder), erectile dysfunction questions based on the Study on the Sexual Life of the Brazilian, by Carmita Abdo (a research that turned into a book, about the Brazilian sexual habits, in a simple and objective language, mentioning various topics like: sexual orientation, erectile dysfunction, orgasm, sexual desire and sexually transmitted diseases and the IPSS (questionnaire that evaluates the urinary symptoms and that can be used on both men and women). In relation to premature ejaculation, it was explained to the interviewee the definition and asked if he has frequent or occasional premature ejaculation.

### Statistical analysis

Descriptive analysis with presentation of absolute and relative frequency tables for categorical variables and dispersion measures for numerical variables.

For comparison of proportions we used the chi-square or the Fisher's exact test, when necessary.

The significance level adopted for the statistical tests was 5%.

## RESULTS

From the 6000 distributed questionnaires, 2575 were excluded, and 3425 were analyzed. 1575 male and 1850 female questionnaires were reviewed. In relation to family income, we found 80% individuals from classes D (between two and four minimum wages) and E (less than two minimum wages).

Regarding the age of the elderly, the average age of respondents was 70 years, 69.8 years among men and 70.1 years among women.

### Sexual Function

**Figure 1:**
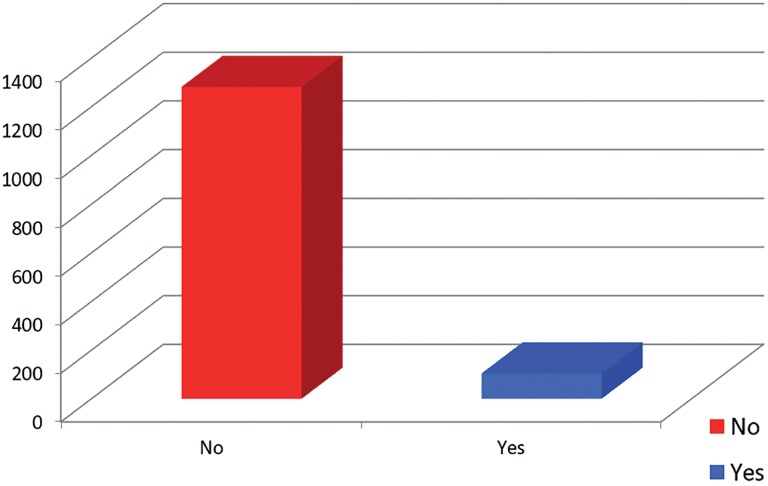
ADAM Questionnaire-Total positive: 92%.

One thousand three hundred and eighty-five ADAM questionnaires were analyzed. In relation to question 1 (reduction in libido) and 7 (erection failure) which separately make up a positive result, we obtained 69% of YES as an answer for question 1 and 79% for question 7. It was observed a total positive of 92%, with a gradual increase according to increase in age (Figure-1).

**Figure 2:**
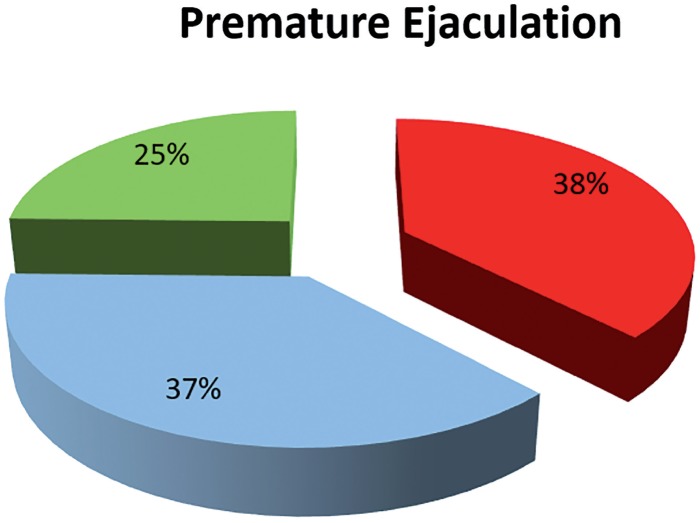
Results of premature ejaculation.

In relation to PE, from the 1220 respondents, 37.5% presented premature ejaculation, and, in addition, 24.7% also occasionally presented PE (Figure-2).

**Figure 3:**
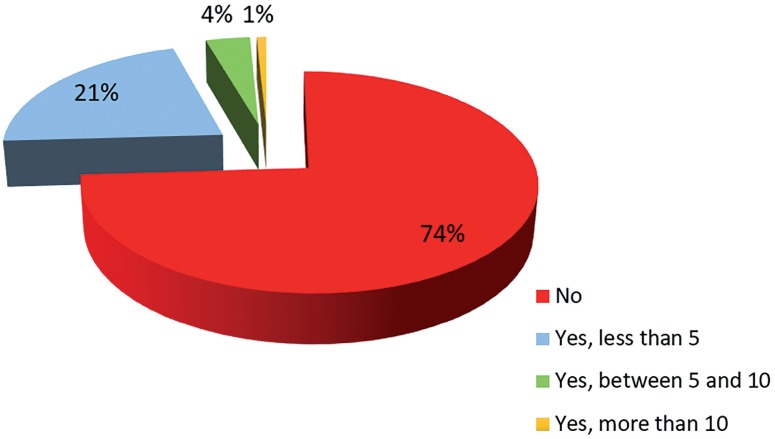
Result of the monthly sexual intercourse of women.

Assessing the number of intercourse in 1755 women, we have identified that more than 90% have none or report to have less than 5 sexual intercourse per month (Figure-3).

The main reasons for the lack of sexual intercourse are: lack of partner and sexual desire. 988 people (78%) answered that they do not want sexual intercourse, and, most important, 272 people (22%) do not have but would like to have sexual intercourse.

### Male IPSS

One thousand four hundred and sixty-five questionnaires were analyzed, with average age of 69.8 years (60-95). The main identified symptoms were respectively: nocturia, frequency lower than 2 hours, urgency, weak stream, incomplete emptying, intermittence and micturition effort, occurring a worsening of the micturition pattern, with more major and moderate symptoms with aging. (Table-1).

**Table 1:** Result of IPSS in men.

IPSS	60-64	65-69	70-74	75-79	≥ 80	Total
**Mild (0-7)**	347 (76.77)	201 (58.43)	183 (62.24)	109 (49.10)	73 (47.71)	913 (62.32)
**Moderate (8-19)**	76 (16.81)	113 (32.85)	86 (29.25)	85 (38.29)	57 (37.25)	417 (28.46)
**Severe (20-35)**	29 (6.42)	30 (8.72)	25 (8.50)	28 (12.61)	23 (15.03)	135 (9.22)
**Total**	**452**	**344**	**294**	**222**	**153**	**1465**

### Female IPSS

One thousand sixty seventy eight questionnaires were analyzed, with average age of 70.1 years (60-99). The main symptoms were, respectively: urgency, frequency lower than 2 hour, nocturia, weak stream, incomplete emptying, intermittence and micturition effort.

Even after 80 years of age, most women showed minor symptoms, with a gradual worsening of the IPSS with aging (Table-2).

**Table 2:** Result of IPSS in women.

IPSS	60-64	65-69	70-74	75-79	≥ 80	Total
Mild (0-7)	366 (69.32)	264 (67.52)	192 (60.95)	133 (56.36)	111 (53.37)	1066 (63.53)
Moderate (8-19)	141 (26.70)	114 (29.16)	99 (31.43)	85 (36.02)	78 (37.50)	517 (30.81)
Severe (20-35)	21 (3.98)	13 (3.32)	24 (7.62)	18 (7.63)	19 (9.13)	95 (5.66)
**Total**	**528**	**391**	**315**	**236**	**208**	**1678**

### Urinary Incontinence

From the 3327 respondents, 853 (25.63%) presented regular leaking of urine, being more common in women than in men (Table-3).

**Table 3:** Prevalence of urinary incontinence.

Incontinence	Woman	Man
No	1194	1280
Yes, 1 or 2 days/week	361	180
Yes, 3 or 4 days/week	90	30
Yes, 5 or more days/week	149	43
**Total**	**1794**	**1533**

Additionally, from the 3347 respondents, 419 (12%) elderly people used some kind of method of protection from leaking in a regular way. Regular usage was observed in 21% of women and 3% of men (Table-4).

**Table 4:** Prevalence of elderly who use methods of protection from urinary leaking.

Use methods of protection	Woman	Man
No	1438	1490
Yes, 1 or 2 days/week	134	22
Yes, 3 or 4 days/week	34	2
Yes, 5 or more days/week	202	25
**Total**	**1808**	**1539**

## DISCUSSION

In Brazil, the statute of the elderly define elderly being the individual aged sixty or over. However, in most other countries, the elderly are those aged sixty-five and over, making it difficult the comparison of papers.

Geriatric disfunctions are under-reported since the elderly are afraid of exposing themselves and being mis-interpreted. Sexual and urinary dysfunctions are associated with many social and psychological losses, with worsened quality of life.

According to data from Brazilian Institute of Geography and Statistics (2013), there are 201 million people living in Brazil, from these, approximately 13% are elderly people. The number of elderly people grows throughout the years, from 2.4% of the Brazilian population in 1940 to 13% in 2013.

In a recent metanalysis that analyzed 76 articles assessing the erectile dysfunction in elderly, it was evidenced that lack of sexual desire was mentioned by 12-51% of the elderly over 60 years, 20-65.9% for over 70 years and 40-82% for over 80 years. Those bothered with erectile dysfunction were 14.3-70% in elderly over than 60 years, 6.7-48% for over 70 years and 38% for over 80 years. And only a small portion of this population seeks medical help or use medicines for the improvement of this problem (13).

The ADAM questionnaire is a screening form to be used in individuals over 40, showing great sensitivity (85%) and specificity (19-60%) and is an acceptable tool to monitor the therapeutic development with hormone replacement. Low specificity can be a result of the high prevalence of psychological symptoms that are not addressed in the questions (14). Furthermore, a correlation was found between having a positive ADAM score and both increased stress levels (p <0.001) and poor sleep quality (p <0.001), with stress displaying the strongest effect (15).

Therefore, there can exist 10.5 million of individuals who present ADAM and are in their homes, without medical follow-up.

Tancredi et al. (16), in relation to the ADAM questionnaire, identified 50% of positive answers for the reduction in libido and 59% in relation to weaker erections. Blumel et al. (14) found in the question about reduction of libido a sensitivity of 63% and specificity of 67% and, in relation to the question about ED, they found a sensitivity of 67% and specificity of 53%. In our work, we found greater positivity in the answers, reflecting a precarious medical assistance provided to the elderly people, especially concerning sexual complaints.

In relation to the sexual function of elderly women, we found in our study 78% of the 1260 with no sexual intercourse and lack of desire, similar to literature, in which 73% presented lack of sexual desire (4). The information of greatest importance is the fact that 22% of these elderly women who have no sexual intercourse, would like to have it. Considering the existence of 15 million elderly women in Brazil, there can be more than 3.2 million elderly women with no sexual intercourse but that would like to have it, and should be better assessed.

In relation to the causes that harm the sexuality of elderly women, many authors have different opinion. Ballone (17) mentions that the main problem for sexual dysfunction in elderly women is dyspareunia. Abdo et al. (4) claims that dyspareunia reduces with aging, having the lack of sexual desire as the main disorder. We have identified dyspareunia as the less common cause for the lack of sexual relation, the main causes are: lack of partner and lack of sexual desire.

We evidenced a predominance of urinary symptoms related to storage, in both sexes. There is a significant worsening in men with aging and in women the minor symptoms prevail in all ages. We should always take into consideration factors of confusion such as: hypertension, diabetes, neurological diseases and use of diuretic.

Rodrigues et al. (18), in a study involving 400 men with BPH, in which 36% of the elderly men were between 60 and 69 years old and 44% being more than 70, presented moderate or major symptoms, similar to what was found in our work. We found 40% of elderly men with IPSS higher than 8, corresponding to approximately 4.6 million of elderly men which will need treatment.

Despite the fact that there are not many studies on female urinary dysfunction, the prevalence of LUTS is 3-23%. The main causes are: stress incontinence (51%), overactive bladder (26%), followed by the difficulty in the urinary emptying (18).

Choi et al. (19), examining the IPSS in 1415 women, found a prevalence in voiding symptoms about 9%, with greater predominance of obstructive symptoms, differently from what was observed in literature and in our work.

Yoo et al. (20), researched the urinary symptoms, through the International Continence Society and the IPSS in the population of South Korea over 40 years old and identified a prevalence of LUTS in 68%, increasing significantly with the years in both men and women. The most common symptoms mentioned were: nocturia, frequency and weak stream. And the IPSS showed that, at least 40%, had moderate symptoms, similar to what was observed in our study.

Concerning OB, a work from college of Porto, found a greater prevalence in men (35%) than in women (29%). In the United States, according to the NOBLE study (21) the global prevalence was similar in men and women (17%). In our work, we found data different from the literature, in which elderly women (28%) showed greater prevalence of OB, corresponding to approximately 4.8 million of elderly women. Despite OB shows greater frequency with aging, it should not be perceived as a part of the aging process, but should be treated instead.

Therefore, there is a great prevalence of OB in the elderly, and, when associated with nocturia, causes several damages such as falls and fractures, with reduction of life span.

Information on UI are divergent and the prevalence in Brazil is almost not mentioned. Menezes et al. (22) found prevalence in non-institutionalized women of 61%. Faria et al. (23), found prevalence of 42% in elderly women. Data on men are scarce and confusing. We found 600 (33%) elderly women and 253 (16%) elderly men who were incontinent.

UI is a distressing and disabling condition, which harms psychological, physical and sexual aspects, becoming an important health problem, due to the social and economic impact on these individual lives.

## CONCLUSIONS

Consequently, due to the great number of sexual and urinary disorders found, there is a need of better implementation of public health measures, improving the service and treatment of those, creating a better quality of life for these group of individuals.
